# Editorial: Reviews in the prevention and early detection of oral cancers

**DOI:** 10.3389/froh.2024.1362945

**Published:** 2024-03-05

**Authors:** Abhimanyu Kumar Jha

**Affiliations:** Department of Biotechnology, Sharda School of Engineering and Technology, Sharda University, Greater Noida, India

**Keywords:** oral squamous cell carcinoma (OSCC), oral potentially malignant disorders (OPMD), toluidine blue (TB), notch signalling pathway, cellular senescence and ageing

**Editorial on the Research Topic** Reviews in the prevention and early detection of oral cancers

Oral Squamous Cell Carcinoma (OSCC) is the most common type of oral cancer, primarily originating from the thin, flat cells lining the oral cavity. Epidemiologically, it is a significant global health concern, with tobacco and alcohol consumption being primary risk factors (1). Prevention strategies emphasize tobacco cessation, limiting alcohol intake, and regular dental check-ups. Early detection through routine oral examinations remains pivotal for improved prognosis and survival rates (2). Within this context we invited our researchers to address Reviews in Prevention and Early Detection of Oral Cancers.

Despite facing challenges, disruptions, and uncertainties, we have assembled a diverse and insightful array of manuscript submissions. Four articles with 17 authors from 4 different countries were published in Frontiers in Oral Health and were featured in a special section on oral cancers. Despite the diversity of this collaborative effort, the contributions are divided into four research areas: (1) screening of oral potentially malignant disorders and experiences in Oceania (2) expression of p53 in lesions positive for toluidine blue (3) studying dietary factors along with plant extracts as potential chemopreventive agents in treatment of oral squamous cell carcinoma; and (4) the significance of cellular senescence in aging as well as the development of oral squamous cell carcinoma.

A comprehensive examination in this realm encompasses the diagnosis of oral potentially malignant disorders (OPMD) with a specific focus on the Oceania region. Rich et al., delve into the intricacies of OPMD diagnosis and management, emphasizing the universal principles that should guide these practices worldwide. However, the unique ethnogeographic characteristics of Oceania, which encompasses diverse populations from the islands of Australia to the vast expanses of Melanesia and Polynesia, present distinct challenges and opportunities. Their review sheds light on the current international trends in OPMD classification and diagnosis, enriched by their on-ground experiences spanning Oceania's varied landscapes and cultures, from bustling cities to secluded villages (Rich et al.).

A parallel avenue of investigation centered on the exploration of diagnostic methods and markers for oral squamous cell carcinoma. Bhalang and Danuthai embarked on a study to discern the efficacy of toluidine blue and vinegar in oral cancer detection. Their research, involving 87 patients suspected of having oral squamous cell carcinoma lesions, applied both agents to the lesions followed by subsequent biopsies. The tissues underwent rigorous histopathological and immunohistochemical evaluations for the tumour marker p53 and the proliferation marker Ki67. The findings illuminated that while toluidine blue demonstrated a sensitivity of 93% but a specificity of only 46%, vinegar exhibited a sensitivity of 85% coupled with a higher specificity of 81%. Furthermore, a notable correlation was discerned between vinegar application and Ki67 expression at the cellular level (*p* = 0.019). However, despite variations in p53 expression among specimens, the association with toluidine blue did not attain statistical significance. This research underscores that while vinegar may have a diminished sensitivity compared to toluidine blue, it holds superior specificity in the realm of oral cancer screening, with its clinical outcomes resonating at the molecular level with Ki67 expression (Bhalang and Danuthai).

Research on the use of plant extracts and dietary variables as chemopreventive agents in the treatment of mouth cancer is rapidly expanding. In their exploration of this field, Kumar and Jha emphasized the value of these organic substances in the fight against OSCC. They stressed that although conventional treatment approaches have advanced, the survival rates for OSCC are still depressingly low. Therefore, it is essential to look for alternate therapeutic approaches. These plant extracts and dietary components are abundant in anti-inflammatory, anti-oxidant, and anticancer qualities. Their complex action affects multiple cellular pathways, such as the Sonic Hedgehog pathway, Akt/mTOR/NF-κB signalling, Hippo-Tafazzin signalling, notch signalling, mitochondrial pathways, and others, that are essential to the development of cancer (Kumar and Jha).

OSCC growth is significantly influenced by aging and the cellular alterations that go along with it. In their detailed analysis of this complex interaction, Niklander et al., provide insight into how cellular senescence contributes to the development of ageing and cancer. Senescence is the state that cells reach when they become older and acquire damage. The senescence-associated secretory phenotype (SASP) is the collective designation for these senescent cells that produce inflammatory chemicals and show altered gene expression patterns. The SASP has been linked to the development of several age-related illnesses, most notably cancer. More specifically, in the case of oral cancer, senescent cells’ creation of an inflammatory milieu inside the tumour microenvironment promotes OSCC's growth and invasiveness. Excitingly, the emergence of senotherapeutics, such as senolytics and senomorphics, presents novel strategies to counteract the detrimental effects of senescent cells and their associated inflammatory cascade. The authors provide an insightful review on cellular senescence, emphasizing its intricate link with inflammation-driven cancer progression, particularly OSCC, and discuss potential clinical interventions (Niklander et al.).

It is clear from reading the aforementioned articles that there are many facets to the field of oral cancer research, necessitating a thorough approach to treatment, detection, and prevention. The studies highlights the significance of customized diagnostic methods, like the careful use of vinegar and toluidine blue, as well as the potential of dietary substances to prevent OSCC. Moreover, the complex interactions that occur between cellular senescence, aging, and the advancement of cancer provide novel targets for treatment approaches. Notwithstanding the useful insights these studies offer, it is crucial to recognize their inherent limitations, which mostly consist of cross-sectional designs and potential biases in participant selection. Notwithstanding these limitations, the body of information gathered from these studies presents encouraging avenues for improving the treatment of oral cancer. It emphasizes the need for continued interdisciplinary research to refine our understanding and interventions, ultimately aiming to improve patient outcomes and reduce the global burden of OSCC.
